# The Dental Register of the West

**Published:** 1859-01

**Authors:** 


*The Dental Register of the West* seems somewhat annoyed that we did not copy from its pages the proceedings of the American Dental Convention, held in August last, at Cincinnati, instead of publishing a summary furnished by a member, made up partly from recollection and partly from advance sheets of the Register, and, we believe, of the reporter, which he obtained. The report of the proceedings published by us, it is true, is not as full as the Register's. But it was the only one that came to us in time for our October number. Indeed, we saw no other report until all the forms but one of the Journal were printed. The advance sheets which the editors of the Register speaks of having sent, have not yet reached us.

The Register further calls attention to what he modestly terms want of manliness on the part of a Journal, in not withdrawing a statement to the effect that the senior editor had accused one of the editors of the Register of making an anonymous attack upon him. We are not aware of having accused either of the editors of the Register of having made such an attack. We merely stated, in noticing an article over the letter W, which appeared some months ago, in a number of this publication, criticising some remarks made by the senior editor, at a meeting of the American Dental Convention, that it is seldom we notice the strictures of anonymous writers, &c. At the time this editorial was written, we had no reason to suppose that either of the editors of the Register was the author of the article in question, as it did not appear in the editorial department, and as there are probably fifty dentists in the United States, the spelling of whose names begin with the letter W., we did not regard this initial as sufficient to fix the authorship upon one of the editors of this work. The authorship was afterwards acknowledged by Dr Watt, but we did not deem it a matter of sufficient importance to recur to the subject again, having said nothing that could be regarded as offensive to any one Had we supposed the author desired us to mention his name, we should not have hesitated a moment to have done so.

